# Mechanisms and therapeutic insights from zebrafish models of wound healing

**DOI:** 10.3389/fcell.2026.1943284

**Published:** 2026-09-03

**Authors:** Chengen Feng, Runxiang Li

**Affiliations:** 1 Experimental Center of Zhongshan Campus of Guangdong Pharmaceutical University, Zhongshan, Guangdong, China; 2 Department of Dermatology, Guangzhou Dermatology Hospital, Guangzhou, Guangdong, China; 3 Institute of Dermatology, Guangzhou Medical University, Guangzhou, Guangdong, China

**Keywords:** drug screening, immune regulation, live imaging, oxidative stress, regeneration, wound healing, zebrafish

## Abstract

Wound healing is essential for tissue integrity, yet chronic wounds remain a significant clinical challenge. The zebrafish (*Danio rerio*) offers unique advantages for studying wound repair: optical transparency enables real-time *in vivo* imaging, robust regenerative capacity allows scarless tissue restoration, and genetic tractability facilitates mechanistic dissection. This review synthesizes recent advances using zebrafish skin, corneal, and fin injury models, highlighting epithelial cell collective migration, the spatiotemporal dynamics of immune cell recruitment and functional conversion, and the biphasic regulatory roles of reactive oxygen species (ROS) and key signaling pathways including Wnt/β-catenin, NF-κB, and VEGF. Advanced imaging techniques—live fluorescence microscopy, polarity-sensitive probes, spectral-domain optical coherence tomography (SD-OCT), and optical coherence tomography angiography (OCTA)—are discussed for their capacity to visualize tissue remodeling and vascular regeneration. The zebrafish platform also serves as a powerful *in vivo* system for screening natural products, nanomaterials, and small molecules with pro-healing properties, with mechanistic insights into how these agents modulate ROS, NF-κB, MAPK, Wnt, and VEGF signaling. Finally, we address translational limitations and outline future directions integrating multi-omics and gene editing to develop novel regenerative therapies.

## Introduction

Wound repair is a fundamental process essential for maintaining tissue integrity following injury, involving a coordinated cascade of hemostasis, inflammation, proliferation, and remodeling. Despite extensive research, the management of chronic wounds, often associated with diabetes and aging, remains a significant clinical challenge due to an incomplete understanding of the underlying cellular and molecular mechanisms (Kim et al., 2023; Lin et al., 2023). Traditional mammalian models, while valuable, are limited by their tissue opacity, high cost, and relatively poor regenerative capacity, which can hinder high-throughput screening and real-time, *in vivo* observation of repair dynamics (Naomi et al., 2021; Nzigou Mombo et al., 2023).

The zebrafish (*Danio rerio*) addresses these limitations through a unique combination of attributes that make it particularly suited for wound healing research. Its optical transparency—from embryos through larval stages to the translucent adult tailfin—permits real-time, high-resolution *in vivo* imaging of cellular behaviors at unprecedented spatiotemporal resolution, enabling direct observation of cell migration, immune recruitment, and vascular regrowth (Greenspan et al., 2024; Santoso et al., 2024; Tan et al., 2025). Zebrafish also possess a remarkable capacity for robust and often scarless regeneration of complex tissues, including the fin, skin, and cornea, providing a unique system to study the interplay between wound healing and epimorphic regeneration (Sehring et al., 2022; Ikkala et al., 2022c). Advanced genetic tools, including CRISPR/Cas9 and a rich repertoire of transgenic reporter lines, allow precise dissection of gene function in the repair process (Sipka et al., 2022; Sommer et al., 2020). Moreover, the cost-effectiveness and high fecundity of zebrafish make them an ideal platform for scalable drug screening and preclinical therapeutic evaluation (Bello et al., 2025; Feng et al., 2025).

In this review, we focus on the latest discoveries using zebrafish models to dissect the cellular and molecular mechanisms of wound healing. We discuss the establishment of various injury models, the dynamic cellular behaviors captured by live imaging, the key signaling pathways involved, and the role of the immune microenvironment. Furthermore, we highlight the application of these models in developing novel therapeutic strategies—including natural products, nanomaterials, and small molecules—with particular emphasis on how these interventions modulate the ROS–NF-κB–MAPK–Wnt–VEGF signaling axis that orchestrates wound repair. We also address the translational limitations of the zebrafish model. By synthesizing these recent advances, this review aims to provide a comprehensive framework for leveraging the zebrafish model to advance both basic and translational wound healing research.

### Establishment and advantages of zebrafish wound models

#### Construction of skin, corneal, and fin injury models

The construction of standardized and reproducible wound models is fundamental to quantitative wound healing research. In zebrafish, several robust methods have been developed to induce skin wounds, each with distinct advantages. A rotary tool method has been established to generate reproducible full-thickness skin wounds in adult zebrafish. This simple, cost-effective technique is highly amenable to high-throughput screening and allows for longitudinal live imaging of re-epithelialization, immune cell dynamics, and vascular regrowth within the same animal (Greenspan et al., 2024). In contrast, laser ablation techniques offer superior precision, enabling the creation of wounds with finely controlled size and depth, which minimizes collateral tissue damage and is ideal for mechanistic studies, such as those investigating spinal cord regeneration (El-Daher et al., 2021; Feng et al., 2025).

The zebrafish cornea, due to its transparency and lack of a tear film, provides an exceptional model for studying corneal wound healing. An ocular abrasion model, typically using a fine burr or a mild chemical agent, inflicts a reproducible epithelial defect while preserving the underlying stroma. This model permits detailed observation of re-epithelialization dynamics via fluorescein staining and has revealed a bilateral cellular response following unilateral injury, highlighting the role of epithelial cell plasticity (Ikkala et al., 2022a; Ikkala et al., 2022b). Pharmacological studies in this model have shown that compounds like polydeoxyribonucleotide (PDRN) can accelerate corneal closure and modulate key molecular markers such as matrix metalloproteinase-9 (Mmp-9) (Edirisinghe et al., 2022).

The fin amputation model is perhaps the most widely used system for studying epimorphic regeneration. Following amputation, a rapid wound epidermis forms, followed by the development of a blastema—a proliferative cell mass that gives rise to the regenerated structures. This model has been instrumental in dissecting the cellular origins of regenerating tissues. For instance, lineage tracing studies have shown that mature osteoblasts near the injury site dedifferentiate and migrate to form the osteogenic blastema, with distinct signaling pathways, such as NF-κB and actomyosin dynamics, regulating dedifferentiation and migration independently (Sehring et al., 2022). The fin model has also been used to demonstrate the universal requirement of Midkine-a for the initial proliferative burst during regeneration and to show how environmental toxins, like triphenyltin chloride, impair regeneration by inhibiting Wnt signaling (Ang et al., 2020; Cheng et al., 2025). See Table 1.

**TABLE 1:** Zebrafish wound healing models, construction methods and core research functions.

Model type	Construction method	Key advantages	Main research contents	Representative references
Adult zebrafish skin wound model	Rotary tool-induced full-thickness skin injury	Simple operation, high reproducibility, suitable for longitudinal live imaging	Re-epithelialization, immune cell dynamics, vascular regeneration	Greenspan et al., 2024
Zebrafish corneal wound model	Fine burr/chemical agent-induced epithelial abrasion	Optical transparency, no tear film interference, clear observation of epithelial repair	Corneal re-epithelialization, epithelial cell plasticity, drug efficacy evaluation	Ikkala et al., 2022a, Ikkala et al., 2022b
Zebrafish caudal fin amputation model	Surgical amputation of larval/adult zebrafish tail fin	Scarless regeneration, obvious blastema formation, convenient quantitative analysis	Epimorphic regeneration, cell dedifferentiation, key signaling pathway regulation	Sehring et al., 2022
Laser ablation injury model	Precise laser-induced local tissue damage	Minimal collateral damage, controllable wound size and depth	Local cell response, spinal cord regeneration, precise molecular mechanism research	El-Daher et al., 2021

This table summarizes four classic zebrafish wound models, including their construction methods, unique advantages, main research directions and representative literature, providing a reference for standardized model selection in wound healing research.

#### Unique advantages of the zebrafish model

The unique advantages of the zebrafish—optical transparency, genetic tractability, and robust regeneration—collectively create a powerful system for wound healing research. Live imaging capabilities are unparalleled, allowing researchers to observe the dynamic behavior of fluorescently labeled cells. For example, studies using polarity-sensitive carbon dot probes have revealed transient increases in cellular polarity at wound margins immediately following injury, providing a new dimension to our understanding of the wound microenvironment (Hu et al., 2021). Genetic tools have been pivotal in establishing causality. For instance, CRISPR/Cas9-mediated knockout of the aryl hydrocarbon receptor (AHR) genes abolished the pro-regenerative effects of Radix Rehmanniae Praeparata, confirming the AHR-dependent mechanism of autophagy in fin regeneration (Chen et al., 2024). The regenerative capacity itself is a major advantage. Zebrafish can regenerate complex tissues with minimal scarring, offering a comparative model to understand why mammals have limited regenerative abilities. The fin epithelium, for example, serves as a reservoir of highly migratory cells that can be rapidly mobilized to expedite wound closure (Santoso et al., 2024). These features make zebrafish an ideal vertebrate model to bridge the gap between *in vitro* assays and mammalian studies. See Supplementary Figure 1.

### Cell behavior and dynamic monitoring in wound healing

#### Epithelial cell migration and regeneration

Epithelial cell migration is the primary driver of re-epithelialization, and zebrafish models have provided unprecedented insights into this process. Live imaging studies have demonstrated that both skin and corneal epithelial cells close wounds primarily through collective migration, a coordinated movement of cell sheets. Remarkably, adult zebrafish can close large cutaneous wounds within hours, with minimal scarring (Santoso et al., 2024). This rapid closure is facilitated by fin-resident epithelial cells (FECs) that are highly motile. These cells exhibit active lamellipodia formation, promoted by extracellular matrix components such as laminins, enabling them to cover large wound areas rapidly (Santoso et al., 2024).

At a cellular level, migrating epithelial cells undergo profound morphological changes, including dynamic remodeling of the actin cytoskeleton to form lamellipodia and filopodia. Particle image velocimetry (PIV) analysis of the zebrafish posterior lateral line primordium has revealed tissue-scale polarity, with leader cells at the migration front exhibiting distinct dynamic protrusions compared to follower cells (Sampedro et al., 2021). Furthermore, bioelectrical cues have been identified as key regulators. Zebrafish basal skin cells can detect changes in transepithelial electrical potential following injury, which triggers actin polarization and directs cell migration even in the absence of osmotic swelling (Kennard and Theriot, 2020). Molecularly, purinergic signaling via ATP and P2Y2 receptors promotes keratinocyte migration and an epithelial-mesenchymal transition (EMT)-like process, while the dual inhibition of TGFβ receptor and ROCK signaling can reverse this EMT, highlighting the plasticity of epithelial cells during migration (Pascual et al., 2021; Pawlowska et al., 2024).

#### Recruitment and functional conversion of immune cells

The innate immune response is critical for clearing debris and orchestrating repair. In zebrafish, neutrophils and macrophages are rapidly recruited to the wound site. Live imaging has revealed that these cells are recruited both locally and from distal hematopoietic tissues, and their resolution occurs predominantly through reverse migration rather than apoptosis (Kaveh et al., 2020). Macrophages, in particular, exhibit remarkable functional plasticity. They can be reprogrammed towards a pro-inflammatory phenotype to enhance bactericidal activity or can transition to an anti-inflammatory, pro-reparative state to support tissue remodeling (López-Cuevas et al., 2024). This phenotypic switch is crucial for the transition from inflammation to regeneration.

The functional importance of macrophages extends beyond immune defense. In a zebrafish muscle regeneration model, a subset of macrophages residing at the injury site secreted nicotinamide phosphoribosyltransferase (NAMPT), which directly acted on muscle stem cells via the Ccr5 receptor to promote their proliferation (Ratnayake et al., 2021). This finding demonstrates that macrophages create a transient niche that actively supports stem cell-driven regeneration. Additionally, the cytoskeletal dynamics of immune cells, particularly microtubule-dependent cell polarity, are essential for their navigation through the densely packed epidermis to reach wound sites and for efficient phagocytosis (Peterman et al., 2025).

### Molecular mechanisms and signal pathway analysis

#### Biphasic ROS signaling in wound repair

Reactive oxygen species (ROS) have emerged as critical signaling molecules, rather than merely damaging byproducts, in wound healing. Zebrafish models have been instrumental in dissecting their nuanced, biphasic roles. Following injury, a rapid and sustained production of hydrogen peroxide (H_2_O_2_) is observed, which acts as a key chemoattractant for immune cells and regulates various cellular processes (Thauvin et al., 2022). At low to moderate levels, ROS promote wound healing by facilitating immune cell recruitment, regulating cell migration, and activating proliferative signaling pathways. Conversely, excessive or sustained ROS production impairs healing by causing oxidative damage and perpetuating inflammation. This biphasic nature—where ROS serve as both essential signaling mediators and potential pathological agents—underscores the need for therapeutic strategies that modulate, rather than eliminate, redox balance.

One crucial function of ROS is the regulation of wound contraction. Studies have shown that ROS modulate the phosphorylation of myosin regulatory light chain (MRLC), a key component of the actomyosin cytoskeleton. Acute inhibition of ROS leads to impaired wound relaxation and delayed closure, while chronic ROS depletion alters tissue stiffness, causing overcontraction (Ding et al., 2025). Thus, ROS act to fine-tune the balance of contractile forces, preventing overcontraction that would impede closure. Furthermore, ROS signaling is tightly integrated with other pathways. An early reciprocal regulatory interaction between Sonic hedgehog (Shh) and H_2_O_2_ establishes a feedback loop essential for fin regeneration, where H_2_O_2_ controls Shh expression and Shh in turn regulates the H_2_O_2_ level via a canonical pathway (Thauvin et al., 2022). The redox environment also influences macrophage polarization, with distinct redox states characterizing pro-inflammatory and anti-inflammatory phenotypes during fin fold regeneration (Paredes et al., 2021). Given the dual role of ROS, therapeutic strategies aim to modulate, not eliminate, them. ROS-scavenging nanomaterials, such as ceria-based nanocomposite hydrogels, have shown promise in maintaining redox balance and enhancing skin wound repair in both zebrafish and mammalian models (Gong et al., 2022; Joorabloo and Liu, 2024).

#### Regulation of inflammatory cytokines

Inflammatory cytokines orchestrate the recruitment and activity of immune cells. In zebrafish wound models, tumor necrosis factor-alpha (TNF-α) and interleukin-6 (IL-6) are rapidly upregulated following injury. Their expression is dynamically regulated; treatment with anti-inflammatory phytocompounds downregulates these cytokines, correlating with reduced neutrophil infiltration and accelerated healing (Vaidyanathan and Lokeswari, 2024). The therapeutic benefit of modulating these cytokines is further supported by studies using antimicrobial peptide-loaded chitosan nanoparticles, which suppress TNF-α expression while enhancing wound closure (Dias et al., 2025).

Heat shock proteins (HSPs), particularly HSP70, are also induced following tissue injury and serve a protective role by stabilizing proteins and modulating immune responses. In zebrafish treated with bacterial extracellular vesicles, HSP70 expression was significantly upregulated alongside pro-inflammatory cytokines, indicating a coordinated stress and immune response (Dias et al., 2024). This interplay suggests that HSPs not only mitigate cellular stress but also actively shape the inflammatory microenvironment to support repair.

#### Angiogenesis and tissue regeneration signaling

Angiogenesis is essential for supplying oxygen and nutrients to the healing wound. Live fluorescence imaging in adult zebrafish has shown that following skin injury, quiescent endothelial cells and pericytes become activated, driving angiogenesis through sprouting, elongation, and anastomosis to form a new vascular network (Fukuhara, 2020; Yuge et al., 2023). Hemodynamic forces, particularly blood flow-driven intraluminal pressure, are critical regulators of this process, influencing endothelial cell proliferation and vessel maturation (Yuge et al., 2023).

The extracellular matrix (ECM) provides critical guidance cues. Disruption of collagen cross-linking or depletion of collagen-producing cells in zebrafish leads to impaired endothelial migration and abnormal vessel morphology, demonstrating that ECM integrity is essential for proper vascular network formation (Senk and Djonov, 2021). Among pro-angiogenic factors, the vascular endothelial growth factor (VEGF) pathway serves as the upstream master regulator. Hypoxia induced by cobalt chloride enhances VEGF expression and neovascularization, accelerating fin regeneration (Rathinasamy et al., 2025). Conversely, inhibition of VEGF receptors impairs angiogenesis. Downstream of and in coordination with VEGF, additional factors contribute to vascular remodeling: Apela, expressed in regenerating fin tissue, facilitates vessel remodeling, and Nephronectin is indispensable for vascularization during fin regeneration by interacting with integrins (Patra et al., 2025; Nys et al., 2024). Understanding this hierarchical organization—VEGF as the primary driver, with Apela and Nephronectin as cooperative co-factors—clarifies how multiple pro-angiogenic signals converge to restore vascular function.

#### Extracellular matrix and migration-promoting factors

The ECM not only provides structural support but also actively promotes cell migration. Laminins, enriched in the fin, promote FEC migration by facilitating lamellipodia formation, accelerating re-epithelialization (Santoso et al., 2024). Cartilage acidic protein 1 (CRTAC1), an ECM protein, has been identified as a potent promoter of fibroblast migration in zebrafish skin models, enhancing motility through actin-driven cytoskeletal reorganization (Félix et al., 2021). Matrix metalloproteinases (MMPs) are crucial for dynamic ECM remodeling. In zebrafish keratocytes, while MMP13 exhibits an anti-migratory effect, MMP2, MMP9, and MMP14 promote migration by regulating ECM turnover. The post-translational activation of MMP2 correlates with localized collagen degradation during fin fold morphogenesis, emphasizing its role in ECM remodeling during regeneration (Wyatt and Crawford, 2021).

### The role of immune regulation and inflammatory response

#### Macrophage polarization and functional conversion

The shift from pro-inflammatory (M1) to anti-inflammatory reparative (M2) macrophages is a critical determinant of healing outcomes. Zebrafish models have been instrumental in visualizing this process and identifying modulators. Dried tangerine peel polysaccharide (DTPP) was shown to promote M2 macrophage polarization and accelerate wound healing in both zebrafish and mouse models of diabetes. Mechanistically, DTPP binds to and inhibits the TLR4/MD-2 complex, thereby attenuating pro-inflammatory signaling and fostering an anti-inflammatory environment (Lin et al., 2024). This study highlights the zebrafish’s utility for screening bioactive compounds that influence macrophage function.

Similarly, carboxymethylated yeast β-glucan (CMG3) exhibits immunomodulatory activities that promote M2 polarization, enhance angiogenesis, and support tissue regeneration in diabetic mice, as demonstrated through initial zebrafish bioactivity screening (Xu et al., 2025). These findings collectively underscore the therapeutic potential of natural products that modulate macrophage polarization to improve wound repair, especially in chronic, inflamed conditions. See Table 2.

**TABLE 2:** Therapeutic agents targeting zebrafish wound healing and their regulatory mechanisms.

Therapeutic agent type	Representative agents	Action target/Pathway	Biological effects on wound healing	Representative references
Natural products	Dried tangerine peel polysaccharide	TLR4/MD-2 complex	Promotes M2 macrophage polarization, accelerates diabetic wound repair	Lin et al., 2024
	Betulin	ROS/MAPKs/NF-κB pathway	Inhibits inflammatory factors, enhances caudal fin regeneration	Ouyang et al., 2022
	Radix rehmanniae praeparata	AHR-dependent autophagy	Upregulates autophagy, promotes fin tissue regeneration	Chen et al., 2024
	Bulbine species extracts	Inflammatory and ECM remodeling signaling	Accelerates fin regeneration, promotes re-epithelialization	Segone et al., 2025
Nanomaterials/biopolymers	β-chitosan/ZnO nanoparticles	Antimicrobial and anti-inflammatory pathways	Antibacterial, reduces inflammation, accelerates epithelialization	Ramachandran et al., 2024
	Octominin-loaded chitosan nanoparticles	Pro-inflammatory cytokine signaling	Inhibits TNF-α/IL-1β, accelerates dermal wound closure	Dias et al., 2025
	Lignin nanoparticles	Wnt/β-catenin pathway	Anti-oxidation, anti-inflammation, activates regeneration	Bragato et al., 2024
​	Therapeutic agents targeting zebrafish wound healing and their regulatory mechanisms Ceria-based nanocomposite hydrogel	ROS scavenging	Maintains redox balance, promotes skin wound repair	Gong et al., 2022
Small molecular compounds	Polydeoxyribonucleotide	MMP-9 signaling	Accelerates corneal wound closure, regulates ECM degradation	Edirisinghe et al., 2022
	Modified ATP analogue	Purinergic receptors	Promotes keratinocyte migration, accelerates tail fin regeneration	Pawlowska et al., 2024

This table classifies wound healing therapeutic agents based on material properties, and clarifies their action targets, phenotypic effects and supporting literature in zebrafish models, providing a basis for translational research of wound repair drugs.

#### Spatiotemporal dynamic regulation of inflammatory cells

The dynamic behavior of inflammatory cells is tightly regulated in space and time. Live imaging has shown that neutrophils infiltrate the wound site within minutes, followed by macrophages. These cells are not static; they exhibit complex behaviors such as reverse migration, which contributes to inflammation resolution (Robertson and Huttenlocher, 2022). The metabolic state of these cells is intimately linked to their function. *In vivo* fluorescence lifetime imaging of autofluorescent metabolic coenzymes NAD(P)H and FAD has revealed that pro-inflammatory macrophages exhibit a more oxidized redox state compared to their anti-inflammatory counterparts during tissue repair (Miskolci et al., 2022). This metabolic rewiring is functional, as glycolysis inhibition alters macrophage redox balance and impairs regeneration.

#### Impact of immune microenvironment on chronic wounds

Chronic wounds are characterized by persistent inflammation and an imbalanced immune microenvironment. Zebrafish models have recapitulated this, showing that sustained neutrophil and macrophage infiltration, coupled with excessive production of TNF-α and IL-1β, contributes to healing impairment (Raziyeva et al., 2021). The failure to appropriately switch from M1 to M2 macrophages is a key feature of this dysregulation, and recent evidence suggests that exosome-induced macrophage reprogramming may offer therapeutic promise for restoring the M1/M2 balance (Wang et al., 2025). Consequently, immunomodulatory strategies have emerged as promising therapies. For instance, hydroxybutyl chitosan/diatom biosilica hydrogels have been developed to induce M2 macrophage polarization, thereby promoting angiogenesis and re-epithelialization in chronic wound models (Cao et al., 2024). These approaches highlight the therapeutic potential of targeting immune cell polarization to resolve chronic inflammation and restore tissue homeostasis.

### Novel therapeutic strategies and drug screening applications

#### Natural products and traditional Chinese herbal medicines

Zebrafish models have been widely used to screen and validate the pro-wound healing effects of natural products. A systematic review identified 38 medicinal plants with promotive effects on zebrafish fin regeneration (Bello et al., 2025). For example, extracts from South African Bulbine species, rich in phenylanthraquinones, demonstrated significant wound-healing properties, with Bulbine frutescens showing a 90% caudal fin regeneration rate (Segone et al., 2025). Betulin, a triterpenoid from birch bark, enhanced fin regeneration by downregulating inflammatory cytokines (IL-1β, TNF-α) and key signaling proteins in the MAPKs/NF-κB pathway (Ouyang et al., 2022). These studies validate traditional uses of medicinal plants and uncover their molecular mechanisms. See Supplementary Figure 2.

#### Application of nanomaterials and biopolymers

Nanomaterials offer a multifaceted approach to wound healing. β-chitosan combined with zinc oxide nanoparticles (ZnO NPs) has demonstrated antimicrobial, anti-inflammatory, and epithelialization-promoting effects in zebrafish models (Ramachandran et al., 2024). Encapsulation of therapeutics within nanoparticles can enhance their efficacy. Octominin-loaded chitosan nanoparticles accelerated dermal wound closure more effectively than the peptide alone while suppressing pro-inflammatory cytokine expression (Dias et al., 2025). Other biopolymers, such as lignin nanoparticles, have shown anti-inflammatory and antioxidant activities, modulating cytokine recruitment and activating regenerative signaling pathways like Wnt/β-catenin (Bragato et al., 2024). These studies highlight the zebrafish model as a powerful *in vivo* platform for evaluating the safety and efficacy of complex nanotherapeutics. See Table 2.

#### Mechanistic links between therapeutics and signaling pathways

A central insight emerging from zebrafish-based therapeutic screens is that the agents described above do not act through isolated mechanisms but rather converge on the ROS–NF-κB–MAPK–Wnt–VEGF signaling axis introduced in Section 3. Understanding these mechanistic links is essential for translating pre-therapeutic efficacy in zebrafish into rational clinical interventions. Ceria-based nanocomposite hydrogels exemplify this principle: cerium oxide nanoparticles cycle between Ce^3+^ and Ce^4+^ oxidation states, mimicking endogenous superoxide dismutase and catalase activities. By decomposing excess H_2_O_2_ and superoxide at the wound site, these hydrogels restore the biphasic ROS balance described in Section 3.1—reducing oxidative damage while preserving the low-level ROS gradient required for neutrophil chemotaxis (Gong et al., 2022; Joorabloo and Liu, 2024). The resulting redox environment favors macrophage M1-to-M2 transition, which in turn releases VEGF and other pro-angiogenic factors that feed back into the signaling network depicted in Supplementary Figure 1C.

Natural products modulate the same cascade at distinct nodes. DTPP binds the TLR4/MD-2 complex, attenuating downstream NF-κB and MAPK activation and thereby reducing TNF-α and IL-6 production; this receptor-level intervention shifts the M1/M2 balance toward M2 polarization (Lin et al., 2024). Betulin acts further upstream by simultaneously inhibiting the ROS/MAPKs/NF-κB axis, effectively breaking the positive-feedback loop between oxidative stress and inflammation that otherwise perpetuates chronic wounds (Ouyang et al., 2022). Lignin nanoparticles activate Wnt/β-catenin signaling, which cross-talks with TGF-β and VEGF pathways to promote blastema formation, fin outgrowth, and vascular regrowth (Bragato et al., 2024). Small molecules such as PDRN act downstream on MMP-9 to accelerate corneal re-epithelialization and ECM remodeling (Edirisinghe et al., 2022), while modified ATP analogues engage purinergic receptors to promote keratinocyte migration and EMT-like conversion (Pawlowska et al., 2024). Together, these studies demonstrate that zebrafish-screened therapeutics engage the core signaling crosstalk at multiple entry points, validating the zebrafish platform not only for phenotypic screening but also for mechanistic dissection of drug action.

#### Drug screening and efficacy evaluation platform

The zebrafish is an ideal platform for high-throughput drug screening. Its transparency allows for real-time, non-invasive visualization of drug effects on cell migration, inflammation, and angiogenesis. Transgenic lines further enhance this capability, enabling the tracking of specific cell types. This platform has been used to screen for compounds that modulate specific phases of wound healing, including in complex conditions such as diabetic wounds where healing is impaired by persistent inflammation (Lin et al., 2023). For instance, ATP analogues targeting purinergic receptors were shown to enhance keratinocyte migration and EMT, with corresponding improvements in zebrafish tail regeneration assays, demonstrating the model’s utility in identifying novel pro-healing compounds (Pawlowska et al., 2024).

#### CRISPR/Cas9-mediated mechanistic validation

The advent of CRISPR/Cas9 has revolutionized gene function studies in zebrafish, enabling precise validation of gene roles in wound healing and regeneration. Precise gene knockouts have been used to confirm the involvement of key genes. For example, knockout of AHR genes abolished the pro-regenerative effects of Radix Rehmanniae Praeparata, confirming its mechanism of action via AHR-dependent autophagy (Chen et al., 2024). Similarly, knockout of dact2 revealed its inhibitory role in EMT during wound re-epithelialization (Kim et al., 2020). Beyond CRISPR, morpholino antisense oligonucleotides (MOs) remain a valuable tool for rapid, transient gene knockdown during early developmental stages, and an expanding repertoire of transgenic reporter lines—such as Tg (mpeg1:EGFP) for macrophage tracking and Tg (fli1:EGFP) for vascular visualization—enables real-time functional readouts. The integration of gene editing with pharmacological interventions allows for the precise dissection of complex regulatory networks and paves the way for identifying patient-specific molecular targets for personalized regenerative medicine (Arjmand et al., 2020).

### Imaging technologies and monitoring methods

#### *In vivo* fluorescence imaging and polarity-sensitive probes

*In vivo* fluorescence imaging is a cornerstone of zebrafish wound healing research. Transgenic lines allow for the real-time visualization of specific cell types, such as neutrophils and macrophages, and molecular events like H_2_O_2_ production (Pham et al., 2020; Sipka et al., 2022). Advanced techniques, such as two-photon microscopy, enable deep-tissue imaging of cytoskeletal dynamics in immune cells with minimal phototoxicity (Williantarra et al., 2024). The development of near-infrared (NIR) polarity-sensitive carbon dots (PPh-CDs) has enabled the dynamic monitoring of cellular polarity at wound sites, revealing a surge in polarity changes immediately following tail amputation, which correlates with the progression of healing (Hu et al., 2021).

#### Structural optical coherence tomography in regeneration research

Beyond fluorescence methods, structural optical coherence tomography (OCT) has emerged as a powerful, non-invasive modality for longitudinal assessment of tissue damage and regeneration in zebrafish. Bailey et al. (2012) first demonstrated that spectral-domain OCT (SD-OCT) can accurately represent retinal lamination and photoreceptor loss and recovery during light-induced damage and subsequent regeneration in adult zebrafish. Their measurements of retinal morphology by SD-OCT closely matched those obtained by histology of the same eyes, establishing SD-OCT as a quantitative, non-terminal method for tracking the same individual through the entire regenerative time course—an advantage unique to transparent zebrafish models.

Building on this foundation, DiCicco et al. (2014) established an OCT-guided laser photocoagulation model that enables precise focal retinal injuries with real-time visualization of lesion application. By using a beam combiner to integrate OCT imaging with 532-nm laser delivery, they created reproducible outer-retina lesions (≈26,000 μm^2^) and followed lesion progression and regeneration over 3 weeks, demonstrating that OCT can serve as both a targeting tool and a longitudinal readout. More recently, Hammer et al. (2022) combined structural OCT with quantitative behavioral assays (optokinetic response and a vision-dependent social preference test) to demonstrate that visual function is gradually restored during retinal regeneration—with OCT confirming the restoration of retinal architecture and behavioral assays revealing that vision for high-contrast stimuli recovers within 7–10 days, while more demanding low-contrast vision recovers only after 14 days.

Together, these studies establish structural OCT as a versatile tool that, complementary to OCTA, enables non-invasive monitoring of tissue remodeling, structural re-establishment, and functional recovery in zebrafish wound healing and regeneration models. Its capacity for repeated, non-terminal imaging makes it particularly valuable for studies requiring within-subject longitudinal comparisons across the full regenerative time course.

#### Optical coherence tomography angiography (OCTA)

OCTA extends structural OCT by providing three-dimensional visualization of microvascular structures and blood flow. In adult zebrafish, OCTA has been used to longitudinally monitor cutaneous wound healing, revealing significant differences in tissue remodeling and angiogenesis between diabetic and non-diabetic wounds (Kim et al., 2023). The diabetic wounds exhibited delayed angiogenesis, which correlated with slower recovery. OCTA has also been integrated with machine learning to automate the quantification of wound morphology and vascular features, enhancing the objectivity and reproducibility of assessments (Wang et al., 2023).

#### Limitations and translational considerations

While the zebrafish model offers remarkable advantages for wound healing research, several important limitations must be acknowledged when translating findings to clinical applications. First, zebrafish lack mammalian skin appendages such as hair follicles, sebaceous glands, and sweat glands, which play significant roles in mammalian wound repair and scarring. Second, the zebrafish coagulation system differs from that of mammals—zebrafish thrombocytes (the equivalent of platelets) have distinct activation mechanisms, and the fibrinolytic pathway operates differently, which may affect the hemostatic phase of wound healing. Third, immune molecule naming conventions differ between species (e.g., mmp9 vs. MMP9 in mammals), and the zebrafish innate immune system predominates during early developmental stages, whereas mammals rely on both innate and adaptive immunity from birth. Fourth, zebrafish are aquatic organisms maintained at 28 °C, and their metabolic rate, tissue turnover, and drug pharmacokinetics differ substantially from those in mammals at 37 °C, potentially complicating dose translation. Finally, the rapid and often scarless regeneration observed in zebrafish may not fully recapitulate the fibrotic healing that characterizes most mammalian wounds. These limitations do not diminish the model’s value for mechanistic discovery and drug screening, but they underscore the necessity of validating zebrafish findings in mammalian models before clinical translation.

## Conclusion

The zebrafish model has established itself as a pivotal platform for advancing our understanding of wound healing mechanisms. Its unique attributes—optical transparency, genetic tractability, robust regenerative capacity, and suitability for high-throughput screening—provide unparalleled opportunities to dissect the complex cellular and molecular events underlying tissue repair in real time. The zebrafish system effectively bridges the gap between *in vitro* studies and mammalian models, providing a dynamic and integrative environment to observe processes such as cell migration, immune modulation, signaling pathway activation, and angiogenesis.

Significant strides have been made in elucidating the fundamental aspects of wound healing, including the rapid collective migration of epithelial cells, the spatiotemporal dynamics of immune cell recruitment and functional conversion, and the biphasic regulatory roles of ROS and inflammatory signaling. The application of cutting-edge technologies, such as CRISPR/Cas9 gene editing, live fluorescence microscopy, spectral-domain OCT, and OCTA, has revolutionized our ability to capture these dynamics with high precision. Furthermore, the zebrafish has proven to be an invaluable preclinical platform for evaluating emerging therapies, including natural products and nanomaterials—with demonstrated efficacy in modulating the ROS–NF-κB–MAPK–Wnt–VEGF signaling axis that orchestrates wound repair.

Looking forward, the integration of multi-omics technologies—genomics, transcriptomics, proteomics, and metabolomics—with advanced imaging and gene editing holds great promise to further unravel the intricate regulatory networks governing wound healing. Such comprehensive analyses will enable a systems-level understanding, fostering the discovery of novel biomarkers and therapeutic targets with higher precision. By continuing to leverage the unique advantages of the zebrafish while acknowledging its translational limitations, the field can drive translational breakthroughs that significantly impact the clinical management of acute and chronic wounds.

## Supplementary material

SUPPLEMENTARY FIGURE 1 Zebrafish models of wound healing and molecular mechanisms. **(A)** Common injury models used in zebrafish wound healing research, including skin wound, corneal wound, caudal fin amputation, and laser ablation. **(B)** The cellular and tissue response cascade following injury, from neutrophil recruitment to scarless regeneration. **(C)** Simplified signaling network highlighting crosstalk among ROS, Wnt/beta-catenin, NF-kappaB, TGF-beta, MAPKs, and VEGF during wound repair. Solid arrows indicate activation or regulation; double-headed arrows indicate crosstalk. This figure is identical to Supplementary Figure 1 in the main manuscript; it is included here as a separate file for clarity during peer review.

SUPPLEMENTARY FIGURE 2 Therapeutic strategies targeting zebrafish wound healing. Wound progression (injury -> oxidative stress -> inflammation -> impaired healing) is modulated by three classes of therapeutics. Natural products (green), nanomaterials/biopolymers (orange), and small molecules (pink) act on distinct molecular targets and produce overlapping but characteristic therapeutic outcomes. DTPP, dried tangerine peel polysaccharide; PDRN, polydeoxyribonucleotide; NPs, nanoparticles. This figure is identical to Supplementary Figure 2 in the main manuscript; it is included here as a separate file for clarity during peer review.
